# Effect of a Flaxseed Lignan Intervention on Circulating Bile Acids in a Placebo-Controlled Randomized, Crossover Trial

**DOI:** 10.3390/nu12061837

**Published:** 2020-06-19

**Authors:** Sandi L. Navarro, Lisa Levy, Keith R. Curtis, Isaac Elkon, Orsalem J. Kahsai, Hamza S. Ammar, Timothy W. Randolph, Natalie N. Hong, Fausto Carnevale Neto, Daniel Raftery, Robert S. Chapkin, Johanna W. Lampe, Meredith A. J. Hullar

**Affiliations:** 1 Division of Public Health Sciences, Fred Hutchinson Cancer Research Center, Seattle, WA 98109, USA; llevy@FredHutch.org (L.L.); krcurtis@fredhutch.org (K.R.C.); isaacelkon@gmail.com (I.E.); okahsai@fredhutch.org (O.J.K.); hammar@fredhutch.org (H.S.A.); trandolp@fredhutch.org (T.W.R.); draftery@uw.edu (D.R.); jlampe@fredhutch.org (J.W.L.); mhullar@fredhutch.org (M.A.J.H.); 2 Northwest Metabolomics Research Center, Department of Anesthesiology and Pain Medicine, University of Washington, Seattle, WA 98109, USA; nngyen10@uw.edu (N.N.H.); fcneto@uw.edu (F.C.N.); 3 Program in Integrative Nutrition & Complex Diseases, Texas A&M University, College Station, TX 77843, USA; r-chapkin@tamu.edu

**Keywords:** lignans, bile acids, dietary intervention, enterolactone, microbiome

## Abstract

Plant lignans and their microbial metabolites, e.g., enterolactone (ENL), may affect bile acid (BA) metabolism through interaction with hepatic receptors. We evaluated the effects of a flaxseed lignan extract (50 mg/day secoisolariciresinol diglucoside) compared to a placebo for 60 days each on plasma BA concentrations in 46 healthy men and women (20–45 years) using samples from a completed randomized, crossover intervention. Twenty BA species were measured in fasting plasma using LC-MS. ENL was measured in 24-h urines by GC-MS. We tested for (a) effects of the intervention on BA concentrations overall and stratified by ENL excretion; and (b) cross-sectional associations between plasma BA and ENL. We also explored the overlap in bacterial metabolism at the genus level and conducted in vitro anaerobic incubations of stool with lignan substrate to identify genes that are enriched in response to lignan metabolism. There were no intervention effects, overall or stratified by ENL at FDR < 0.05. In the cross-sectional analysis, irrespective of treatment, five secondary BAs were associated with ENL excretion (FDR < 0.05). In vitro analyses showed positive associations between ENL production and bacterial gene expression of the bile acid-inducible gene cluster and hydroxysteroid dehydrogenases. These data suggest overlap in community bacterial metabolism of secondary BA and ENL.

## 1. Introduction

Diets higher in fiber are associated with reduced risk of several chronic diseases, including many cancers, cardiovascular disease, and obesity and related metabolic diseases [1,2,3,4,5]. Health benefits of high-fiber foods may be attributed, in part, to microbial metabolites of plant lignans. Lignans are polyphenols found in a variety of plant foods, including seeds, whole grains, legumes, fruits, and vegetables. They can be converted to enterolignans (enterolactone (ENL) and enterodiol (END)) through gut microbial metabolism [6]. The enterolignans, and ENL in particular, possess a range of biologic activities including anti-proliferative and anti-inflammatory effects, and modulation of estrogen signaling, lipid metabolism, and bile acid regulation [7]. High inter-individual variation in metabolism of lignans has been observed under controlled conditions [8,9], and may influence the level of exposure to the bioactive microbial metabolites and subsequent health-related outcomes.

Bile acids, derived from cholesterol in the liver, are released into the gut lumen in response to ingestion of dietary fat. In the lower gastrointestinal tract, primary bile acids undergo microbial metabolism to secondary bile acids, which have been positively associated with chronic disease, e.g., liver disease and colorectal cancer [10,11]. There is growing recognition that both primary and secondary bile acids are strong signaling molecules involved in metabolism. In the liver, intestine, and other tissues, bile acids interact with the farnesoid-X receptor (FXR) and G-protein-coupled bile acid receptor, more commonly known as TGR5, which contribute to glucose homeostasis and immune regulation [12,13]. ENL interacts with nuclear receptors involved in bile acid metabolism and therefore may mediate bile acid synthesis and metabolism [14]. These interactions between ENL and hepatic receptors, and subsequent microbial activity, lead to a variety of secondary bile acid species [15], and contribute to changes in bile acid pool size and composition.

Given the observation that ENL may affect synthesis and metabolism of bile acids, thereby regulating downstream metabolic effects of bile acids, the primary goal of this analysis was to examine the effects of a lignan intervention on plasma bile acid concentrations. Further, we determined the association between plasma bile acids and ENL excretion cross-sectionally. We also conducted an exploratory analysis, to evaluate whether there was overlap in metabolism at the taxon level. We complemented our analyses with in vitro anaerobic incubations of stool with lignan substrate to identify genes that are enriched in response to lignan metabolism.

## 2. Materials and Methods 

### 2.1. Research Design

Data and biologic samples for the present analysis were derived from the FlaxFX study, a randomized, double-blind, placebo-controlled crossover intervention comparing supplemental flaxseed lignan extract to a placebo [16] (Figure S1). Each intervention period lasted 60 days with at least a 60-day washout period between the two interventions. All study procedures and materials were approved by the Fred Hutchinson Cancer Research Center (Fred Hutch) Institutional Review Board, and informed, written consent was obtained from all participants prior to their starting the study. The study was registered at http://www.clinicaltrials.gov as NCT01619020.

### 2.2. Participants

Details regarding recruitment have been published previously [16]. Briefly, healthy men and women, aged 20 to 45 years, were recruited from the greater Seattle area between September 2012 and August 2016. Exclusion criteria included age <20 or >45 years, tobacco use, consumption of >2 alcoholic beverages/day (equivalent to 720 mL beer, 240 mL wine, or 90 mL hard liquor), regular use of prescription or over-the-counter medications, oral or IV antibiotic use within the past 3 months, weight loss or gain of >4.5 kg in the past year, current or planned pregnancy, breastfeeding, chronic medical illness, history of gastrointestinal disorder and cancer (other than non-melanoma skin cancer), known allergy to nuts, seeds, and flaxseed, inability to swallow pills, and dietary fiber intake ≥20 g/day as assessed using the Block Fruit/Vegetable/Fiber Screener (Nutrition Quest, Berkeley, CA, USA) [17]. The rationale for excluding individuals with higher fiber intakes was to reduce exposure to other plant lignans associated with intake of high-fiber foods.

Participants who met the initial eligibility criteria completed a self-administered food frequency questionnaire [18] and a health and demographic survey, and provided a stool sample. After completing the stool collection, prospective participants also consumed the study flaxseed lignan extract for 3 days and completed a 24-h urine collection on the third day in order to characterize participants as low- or high-ENL excreters for randomization. Individuals still interested in participating in the intervention underwent further screening before randomization, including a medical history, measurement of blood pressure and complete blood count, liver panel, chemistry panel, blood urea nitrogen, serum creatinine measured in a fasting blood draw, and a urine pregnancy test in women [16].

### 2.3. Flaxseed Lignan Extract Supplement

Eligible participants were randomly assigned, blocked on sex and lignan-metabolism status (i.e., ratio of ENL/ (secoisolariciresinol (SECO) + END + ENL) in 24-h urine after 3 days of a daily lignan capsule), to the order in which they received the lignan extract or placebo. Participants consumed the lignan extract capsule containing 50 mg secoisolariciresinol diglycoside (SDG) plus rice bran (Barlene’s Organic Oils, Ferndale, WA, USA), or a visually identical placebo (only rice bran), as one capsule by mouth daily for 60 days. Capsules were packaged by the manufacturer in sealed plastic bottles with a count of 70 capsules per bottle, with all active capsules from the same lot. Separate testing of the lignan extract in triplicate by HPLC [19] confirmed that the mean SDG content was within specifications. The mean lariciresinol and pinoresinol (other plant lignans) content was 0.8 and 3.0 mg/capsule, respectively. During each intervention period, adherence to the study capsules was monitored by pill count. Compliance based on capsules returned was 98% with only one participant falling under 80% compliance during one study period (62% capsules returned during placebo period).

### 2.4. Specimen Collection

Fasting blood samples were collected at the baseline and at the end of each period after a 12-h overnight fast. Blood was collected into vacutainer tubes containing EDTA and was processed and stored as plasma at −80 °C using a standard protocol. Participants collected 24-h urine and stool samples at the beginning and end of each intervention period. Stool samples were collected into RNAlater for bacterial measures as described [16].

### 2.5. Laboratory Analyses

#### 2.5.1. Plasma Bile Acids

Bile acid concentrations were carried out by liquid chromatography tandem mass spectrometry (LC-MS/MS) as previously described [20]. Briefly, 200 μL plasma were combined with 600 μL HPLC-grade methanol and 10 μL solution containing 10 μM of each stable-isotope labeled internal standard (d4-IS): Cholic acid–2,2,4,4–D4 (CA-D4), deoxycholic acid–2,2,4,4–D4 (DCA-D4), glycocholic acid–2,2,4,4–D4 (GCA-D4), and glycochenodeoxycholic acid–2,2,4,4–D4 (GCDCA-D4). The samples were vortexed for 10 s, stored for 20 min at −20 °C. After centrifuge at 18,000× *g* for 15 min (4 °C), 650 μL supernatant was dried at 30 °C in a Speed-Vac for 3 h. The samples were reconstituted in MeOH/H_2_O (1:1, v/v) to 100 μL and centrifuged at 18,000× *g* for 5 min (4 °C). Ninety µL aliquots were transferred into LC vials prior MS analysis. Chromatographic separation was performed using a Waters Acquity I-Class system and a Waters X Select HSS T3 column (2.5 µm, 2.1 × 150 mm). The mobile phase A was 5 mM ammonium acetate in H_2_O, and mobile phase B was acetonitrile, both containing 0.1% acetic acid. Gradient elution mode was as follows: 0.0–1.0 min, 75% A; 1.0–15.0 min, 5% A; 15.0–25.0 min, 5% A; 25.0–25.1 min, 75% A, 25.1–40.0 min 75% A. The flow rate was 0.3 mL/min, column temperature 40 °C, and injection volume 2 μL. Data were measured on a Waters Xevo TQ-S micro mass spectrometer equipped with an electrospray ionization (ESI) source. The ESI conditions were as follows: Electrospray negative ionization mode; voltage 2.0 kV; cone voltage 30 V; source offset 50 V; desolvation temperature 600 °C; cone gas flow 150 L·h^−1^; desolvation gas flow 1000 L·h^−1^. Mass measures of fifty-five bile acids and the d4-IS were recorded using the multiple-reaction-monitoring (MRM) mode. The peak areas were extracted from MRM peaks using Skyline software version 4.2.0. [21]. Absolute bile acid concentrations were calculated using the ratio between the peak areas of each bile acid and the d4-IS and the slope of the calibration curve built with bile acids standard compounds [22,23]. Intra- and inter-assay CVs were 8.7 and 9.8, respectively.

#### 2.5.2. In Vitro Incubations

All participants were offered the opportunity to provide a fresh stool sample on the day of their first blood draw for in vitro analysis. A subset of 9 participants were willing and able to provide a sample. The stool was collected in a plastic tub, and within 30 min, brought into a Bactron Anaerobic Chamber. Inside the chamber the stool was weighed and combined with anaerobic TCAP2 media (Table S1) to a final concentration of 1.66% wt:vol. The stool was broken apart with a spatula and further mixed with a stir bar and magnetic stir plate. The fecal suspension was then filtered through sterile cheesecloth and electronically pipetted into 50 mL glass serum bottles in triplicate (20 mL aliquots). The bottles were inoculated with SDG dissolved in 100% methanol (Thermo Fisher Scientific, Waltham, MA, USA) to a final concentration of 6.55 µM SDG or left un-spiked (blank “control”). The bottles were capped with rubber stoppers and fitted with aluminum seals. The bottle headspace was replaced with 100% high-purity N_2_ for 10 min at the rate of 0.5–1.0 L/min. Bottles were placed in a rotating incubator (C24 Incubator, New Brunswick Scientific, Enfield, CT, USA) and incubated at 37 °C/300 rpm for 6–7 days.

Daily aliquots of fecal suspensions from individual incubating serum bottles were taken at approximately the same time every day using the anaerobic technique and stored at −20 °C in aliquots of 500 µL/day for lignan analysis and 100 µL/day for bacterial enumeration. Samples for DNA analysis were taken on Day 1 and 24 h of the incubation, and stored at −80 °C. The Day 1 sample was two pea-sized scoops of fresh stool dispersed in 5 mL sterile RNAlater (Thermo Fisher Scientific, Waltham, MA, USA) collected in the anaerobic chamber during the previously described procedure. To collect the 24 h incubation pellet, fecal suspensions from serum bottles were combined in a sterile 50 mL centrifuge tube and spun down in a Beckman Coulter Centrifuge at 30× *g*/4 °C for 10 min, supernatant was discarded, and the pellet was re-suspended with 5 mL sterile RNAlater for storage. 

#### 2.5.3. Lignans

SECO, END, and ENL were measured by gas chromatography–mass spectrometry in 24-h urine samples collected at the end of each intervention period [16]. The lowest level of quantitation of the 3 analytes in 2 mL urine was 6.5 ng/mL. The mean intra- and inter-batch coefficients of variation for quality control samples were 5.1 and 9.5% for SECO, 8.0 and 11.3% for END, and 4.8 and 6.0% for ENL, respectively. The same method was used to measure these compounds in the in vitro incubations. For each time point, the measured metabolite concentration was divided by inoculated substrate concentration to calculate percent conversion.

#### 2.5.4. Fecal Microbiome Nucleic Acid Extraction

Stool samples from the intervention collected in RNAlater were thawed and homogenized and two subsamples were extracted for DNA following previously published protocols [24]. We also extracted RNA and DNA from both the 24-h of the in vitro incubations and the stool used as an inoculum for the incubations. Total RNA was extracted [25,26] after the addition of synthetic internal standards [25] and bacterial mRNA was enriched [26,27,28]. The quality of the RNA was evaluated using the Agilent 2100 Bioanalyzer (Agilent Technologies). mRNA amplification was performed using MessageAmpII (Ambion, Foster City, CA, USA). cDNA was generated after addition of an in vitro transcribed internal standard [29].

##### 2.5.5. 16S rRNA Gene Sequencing

Participant stool samples from the baseline and end of each intervention period were analyzed by paired-end sequencing of the V1–V3 region of the 16S rRNA gene, using the 27F mod forward PCR primer sequence 5′-AGRGTTNGATCMTGGCTYAG-3′ and the 519R reverse PCR primer sequence 5′-GTNTTACNGCGGCKGCTG-3′ [30] as described [24]. Sequencing was performed (Molecular Research, Shallowater, TX, USA) on the MiSeq using the MiSeq Reagent Kit v3 following the manufacturer’s guidelines to obtain 2 × 300 bp paired-end reads (Illumina, San Diego, CA, USA). FastQ files were exported and securely transferred to Fred Hutch (BaseSpace, Illumina) for bioinformatic analysis. To classify bacterial taxonomy, sequences were processed using QIIME v 1.9, as previously published, except that we used SILVA (release 132), and OTU picking was implemented in Vsearch [16,24,31,32].

##### 2.5.6. Stool and In Vitro Incubation Metagenomic and Metatranscriptomic Bioinformatic Analysis

Sequence reads were processed for bioinformatic analysis of the metagenomes and metatranscriptomes with the KneadData v 0.5.1 quality control pipeline, which uses Trimmomatic (version 0.36), BMTagger filtering, and decontamination algorithms to remove low-quality read bases and host (human) reads, respectively [33]. Trimmomatic was run with parameters MAXINFO:80:0.5 and MINLEN:50. Functional profiling was performed using HUMAnN2 version 0.11.2 [34] with reads de-paired and implementing Diamond [35] to map reads against UniRef90 [36]. Sequences per gene family were counted, normalized for length and alignment quality, and linked to pathways using MetaCyc [37]. For each participant, data matrices of the abundance of genes, gene families, and genes in metabolic pathways were evaluated. 

#### 2.6. Statistical Analysis

Of the 55 bile acids assayed, 20 had sufficient concentrations to be reliably quantitated and retained for analysis. For one individual, all three time points for muricholic acid were below the limit of detection. These observations were imputed with the equivalent of half the lowest concentration for this bile acid (0.25 nM). A very small value (1 × 10^−4^) was imputed for zero values for ENL (a total of 4). ENL was used for stratified analyses (below and above median: 22.1 µmol/24 h) and association analyses, as it was found to be the most informative metabolite in the parent study [16]. Plasma bile acids and urinary ENL were transformed using the natural logarithm to improve the normality of distributions prior to analysis. 

Linear mixed models were used to (a) test the effects of the flaxseed intervention on individual bile acid concentrations; bile acid groups based on summing individual bile acids into categories of primary, secondary, glycine-conjugated, and taurine-conjugated; and stratified by low- and high-ENL excreters, as determined by below and above median excretion after the flaxseed extract intervention; and (b) cross-sectionally determine the association between plasma bile acids and ENL. Analyses were adjusted for age, sex, body mass index (BMI; kg/m^2^), intervention sequence, assay batch, and baseline bile acid concentrations. One outlier with high baseline values for most bile acids was excluded from intervention analyses but included in all cross-sectional analyses where the baseline was not included in the model. Because dietary fat may affect bile acid concentrations, we further evaluated the inclusion of mean dietary fat intakes at baseline. As point estimates did not differ, results are presented without its inclusion. Potential carryover effects were assessed by including terms for the intervention sequence and interaction of the intervention period. These terms were not found to be significant in any of the models. Cross-sectional analyses were additionally adjusted for intervention and were not adjusted for baseline bile acid concentrations or the intervention sequence. All associations were controlled for multiple testing using the Benjamini–Hochberg algorithm [38], and false discovery rate (FDR) < 0.05 was considered significant.

There is a considerable overlap in the enzymatic reactions involved in metabolism of both lignans and secondary bile acids, i.e., hydroxylation/dehydroxylation, epimerization, and deconjugation reactions [39,40]. Given the association between ENL and bile acids irrespective of treatment, we conducted an exploratory analysis examining the association between the gut microbial composition at the end of placebo, representing an individual’s usual gut microbial composition, and 24-h urinary ENL excretion and individual bile acids at the end of the lignan intervention. A less conservative FDR of 10% was used for this analysis. Files of sequence counts for the gut microbiome were used in statistical analysis. To account for the compositional nature of the microbial genera abundances (*n* = 147), we calculated the centered log-ratio (CLR) transformation [41]. Microbial DNA extracted from two participants did not pass quality control, leaving a sample of *n* = 44 for this analysis. All analyses were performed using Stata (StataCorp v16, College Station, TX, USA). 

The metagenomic and metatranscriptomic sequencing of the in vitro samples resulted in an average of 8.8 (3.5) and 9.4 (2.7 M) sequences before quality control (QC) and 8.8 (3.5) and 9.2 (2.7 M) after QC. The average read length was 142 bp (5). We analyzed both genes and transcripts of genes involved in secondary bile acid production in the stool and in vitro incubations of individuals that produce ENL. Metagenomic and metatranscriptomic samples were assessed at 24 h of the in vitro incubations and in stool samples used for the inoculum in the incubations. An overview of the relative abundance of genes and transcripts was expressed as a relative percent of the total number of secondary bile acid genes in the stool or in vitro samples. To normalize gene expression, we used the ratio of reads per thousand (RPK) counts of metatranscriptome to metagenomic sequences (RNA/DNA) from enzymes involved in secondary bile acid metabolism including 7 α and β hydroxysteroid dehydrogenase (DHSH), 12 α DHSH, and the bile acid-inducible (bai) gene cluster (summed across all genes in the cluster) (Table 1). Further, we assessed the relationship between the bacterial expression of genes involved in secondary bile acids in the in vitro assays and gene expression of genes involved in secondary bile acid production with baseline measurements of SECO, ENL, and END from the sample participants. 

## 3. Results

**Figure 1:**
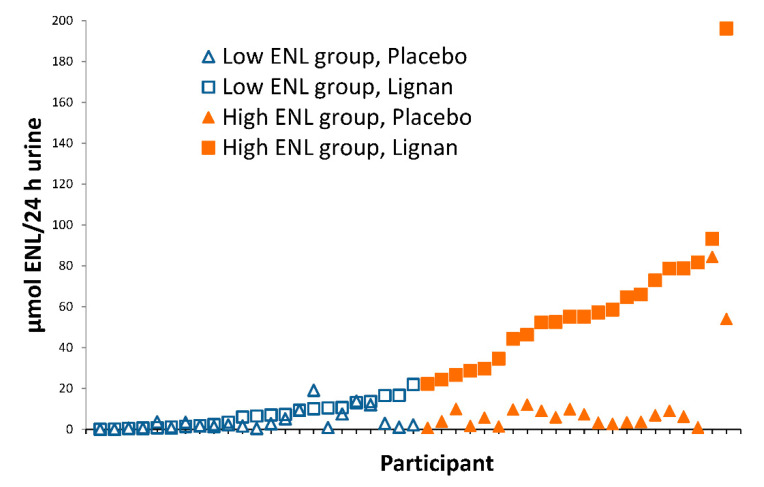
Urinary ENL excretion (24 h) in participants at the end of 60-day placebo (triangles) and lignan (squares) intervention periods. Participants (*n* = 45) were categorized as low (open symbols) and high (solid symbols) ENL excreters based on median ENL excretion after lignan supplementation (22.1 µmol/24 h).

Characteristics of the 46 participants overall and stratified by ENL excretion are given in Table 2. Individual 24 h urinary ENL excretion at the end of the lignan intervention period varied among participants, ranging from 0.8–196 µmol/24 h (Figure 1).

Overall, there were no differences in plasma bile acids after the lignan intervention after controlling for FDR < 0.05; however, mean cholic acid (CA) concentrations tended to be lower after the lignan intervention compared to the placebo (by 46%; *P* = 0.004; Table 3). Similarly, when evaluating the effects of the intervention stratified by low- and high-ENL excreter status, there were no bile acids that differed at FDR < 0.05, although mean glycoursodeoxycholic acid (GUDCA) and glycohyocholic acid (GHCA) was lower in the high-ENL excreter group, taurolithocholic acid (TLCA) was higher among low-ENL excreters, and CA was lower in both groups (*P* < 0.05 for all; Table 3). In the cross-sectional analysis, irrespective of treatment, five secondary bile acids were significantly associated with ENL (FDR < 0.05; Table 4), with one positive association: Isolithocholic acid (ILCA), and four inverse associations: GUDCA, glycohyodeoxycholic (GHDCA), hyodeoxycholic (HDCA), and muricholic acid (MA).

In order to further investigate the role of the gut microbiome, and in particular the overlap between gut bacterial genera associated with ENL and with bile acids, we explored potential associations between the 147 genera in stool at the end of the placebo period and a) urinary ENL excretion and b) the 20 plasma bile acids after the flaxseed lignan intervention. For this analysis, we used an FDR cutoff of 0.1. Although no genera passed the FDR cutoff, four exhibited positive coefficients in the model with ENL *(Alistipes, Ruminococcus_2*, and a bacterium in the Order *Rhodospirallales)* while *Coprococcus_3* suggested an inverse association (Table S2). A total of 65 genera/bile acid associations exhibited a raw *P* value < 0.01 and two secondary bile acids (GHDCA and GUDCA acid) were inversely associated with *Ruminiclostridium_6* at FDR < 0.1 (Table S2).

**Figure 2:**
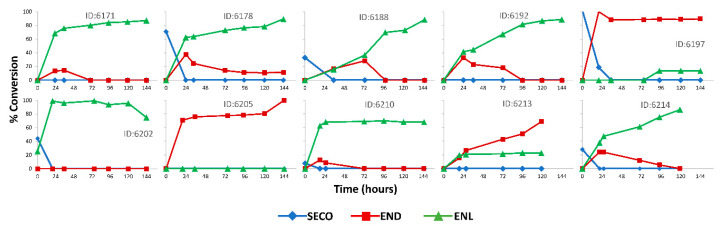
Inter-individual variation in the microbial metabolism of SDG to SECO, END, and ENL in in vitro incubations. Stool samples at baseline were used as an inoculum. Data are presented as mean percent conversion of SDG measured by GC-MS from daily aliquots of fecal suspension over 6 days of SDG-inoculated incubations from participants.

**Figure 3:**
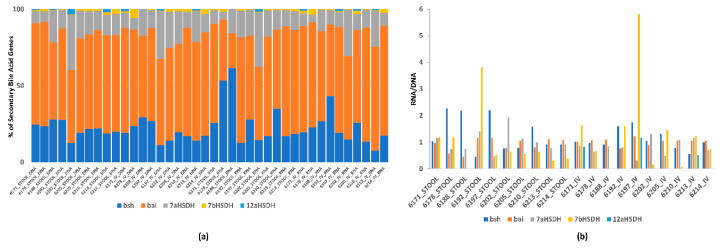
(**a**) The relative percent of bacterial genes and transcripts in the secondary bile acid pathway in ENL producing stool and in vitro (IV) incubation samples (enzyme abbreviations in Table 1). (**b**) Gene expression of bacterial enzymes normalized to gene content (RNA/DNA) involved in secondary bile acid metabolism in ENL producing stool and in vitro (IV) incubation samples (enzyme abbreviations in Table 1).

The stool microbiome capacity for in vitro conversion of SDG to ENL varied across participants (Figure 2). A microbiome that produced ENL was also enriched in transcripts for enzymes involved in secondary bile acid metabolism and gene expression was more variable than gene content (Figure 3a). Although bile salt hydrolase, the *bai* gene cluster and 7α DHSH were present in all samples (Figure 3a), and expression varied (Figure 3b). In contrast, 7β DHSH and 12α DHSH were not detected in all samples, but 7β DHSH showed enriched expression in more of the in vitro samples (5 out of 9) which produced ENL (Figure 3a,b). There was high inter-individual variability in the different types of hydrolases involved in speciation of the secondary bile acids (Figure 3b).

## 4. Discussion

In this ancillary study using data from a randomized, crossover trial, we found modest intervention effects of a flaxseed lignan extract as compared to the placebo on circulating bile acids, mainly CA, overall and by ENL excreter status. However, these effects did not remain significant after controlled for multiple testing. In the cross-sectional analysis, irrespective of treatment, we observed robust associations between ENL excretion and certain circulating secondary bile acids, particularly secondary CA-derived species. These results likely reflect lower liver-derived CA available as substrate.

To date, evaluation of the effects of lignans on bile acids has only been conducted in animal models, mainly in the context of cholestasis [42,43,44,45,46] or hepatocarcinogenesis [47,48]. In vitro, ENL has been shown to inhibit cholesterol 7α hydroxylase (CYP7A1) activity, the enzyme involved in catalyzing the rate-limiting step in the conversion of cholesterol to primary bile acids, [49]. In mice, lignans downregulate liver-X receptor (LXR), the receptor regulating CYP7A1, through an AMP-activated protein kinase-associated mechanism [50,51]. As a moderate activator of pregnane-X receptor (PXR), ENL ligand-binding leads to differential hepatic oxidation and detoxification of secondary bile acids, such as LCA and DCA [52,53]. Bile acids are strong signaling molecules which activate FXR and TGR5 and contribute to the regulation of several systemic endocrine functions, including lipid and glucose metabolism, immune responses, and energy metabolism [54]. Activation of FXR varies, with chenodeoxycholic acid (CDCA) having the greatest potency, followed by LCA, DCA, and finally CA [54]. Conversely, TGR5 is mainly activated by the secondary bile acids, LCA and TLCA [54]. Bile acids can also have pathologic effects in higher concentrations, particularly species with higher hydrophobicity [55]. Therefore, changes in the composition of the bile acid pool may impact health-related outcomes.

In the cross-sectional analysis, irrespective of treatment, there were strong associations between ENL excretion and several secondary bile acid species, particularly CA-derived species which tended to be consistent across analyses. For instance, GUDCA, GHDCA, and HDCA, were inversely associated with ENL, while isolithocholic acid (ICA) was positively associated. GUDCA and GHCA were also the two secondary bile acids that tended to be lower among high-ENL excreters with lignan supplementation. These associations likely reflect lower CA availability. The branch point between the production of CA versus CDCA requires the addition of a single hydroxyl group at the C–12 position of 7α-hydroxy–4–cholestene–3–one, catalyzed by the enzyme CYP8B1 [56]. This reaction leads to the production of CA and controls the ratio of CA to CDCA. Phytochemicals can have a variety of effects through several mechanisms, and numerous compounds have been shown to modulate cytochrome P450 enzymes. For example, a study in mice showed that ellagic acid (a dilactone of hexahydroxydiphenic acid) inhibited CYP8B1 mRNA 8-fold [57]. Thus, it is plausible that ENL is modulating this CYP enzyme, leading to lower production of CA.

Much attention has been given to defining the hepatic biosynthesis of the primary bile acids CDCA and CA [58] and the 7α-dehydroxylation of primary bile acids by bacteria with bai genes to secondary bile acids, LCA and DCA [59,60]. Tertiary bile acid metabolism (i.e., further conversion of secondary bile acids to additional metabolites in the liver) includes two pathways, 6*α*-hydroxylation of LCA to HDCA catalyzed by CYP3A [61], and DCA conversion to HDCA via CYP3A6/3A7 [62]. If ENL down-regulates the amount of substrate converted to CA and CDCA, then the pools available for conversion to tertiary HDCA would also be reduced. Moreover, detectable differences in glycine, but not taurine, conjugates may be due to low circulating concentrations of taurine conjugates. Taurine conjugates constituted roughly 11% of the bile acid pool in our data.

The bile acid composition of the human intestine is determined by multiple factors, including the characteristics of the bioconversion reactions, the abundances of the bacterial taxa involved in the reactions, and the availability of substrates/cofactors in the intestinal environment [63]. Our data suggests that bacteria associated with fiber metabolism may impact secondary bile acid metabolism. *Alistipes* and *Ruminococcus* were positively associated with ENL at *P* < 0.01. These organisms are enriched in genes for complex glycan metabolism or fermentation to short-chain fatty acids associated with fiber metabolism [64]. Although they have not be identified in ENL production per se [65,66], they may be indicative of a habitual fiber-rich diet that would contain more lignan substrates. GUDCA and GHDCA were inversely associated with *Ruminiclostridium_6* at FDR < 0.1. *Ruminiclostridium* is also associated with dietary fiber degradation which may impact the overall pool of bile acids available for conversion to secondary bile acids [67,68]. There were some genera that were correlated with both ENL and secondary or tertiary bile acids. For example, *Coprococcus* was positively associated with ENL and HDCA and inversely with GHCA and tauro-α-MA, whereas *Ruminococcus_2* was inversely associated with ENL and MA. These associations suggest that the microbiome may impact host exposures by altering the speciation of both secondary and tertiary bile acids and ENL.

Inter-individual variation in microbial transformation of lignans and bile acids is influenced by variation in microbial gene content, strain variation in enzyme kinetics, and substrate availability [69]. The complete metabolism of lignans and bile acids to ENL and secondary bile acid species, respectively, can occur in a single organism with the complete metabolic pathway or in a consortia of bacteria whose genome content completes the full metabolic pathway [39,70]. The large number of bacteria that expressed bile salt hydrolase (BSH) in stool or the in vitro incubations suggests that this is not a rate limiting step in secondary bile acid production [71]. However, the high inter-individual variation in the BSH and dehydrogenases suggests that the gut microbiome may influence inter-individual variation in the speciation of secondary bile acids. The positive association between ENL production and gene expression of the bai cluster and dehydrogenases suggests overlap in community metabolism of secondary bile acids and ENL. Additionally, gene expression for secondary bile acid metabolism was enriched in the ENL, producing in vitro incubations. We identified *Eggerthella lenta and Gordonibacter pamelaceae* in the in vitro incubations. Both of these organisms not only have a complete bai gene cluster which is involved in the conversion of primary to secondary bile acids, but they have recently been identified as part of a consortium of bacteria that produce ENL [69,70]. Our previous work showed that the microbial composition at the baseline was positively associated with ENL production in this cohort, reinforcing that the microbial community involved in ENL production may impact secondary bile acid production [16].

To our knowledge, this is the first study to evaluate the effects of lignans on circulating bile acids in humans. Strengths of the study include the robust randomized, crossover design with placebo control, the measurement of a broad range of bile acid species, and the conservative analysis with stringent correction for multiple testing. There are limitations worth noting. We lacked sufficient power to detect significant effects of the lignan flaxseed intervention on many of the bile acids, and concentrations of many bile acid species were below the limit of detection, particularly the taurine-conjugated species, which are typically present in very low concentrations. Post-hoc power calculations using data from this pilot study suggest that the minimum sample size needed to detect a similar effect size for any of the 20 bile acids is 52 for CA, the primary bile acid which had the largest difference between interventions (46%; 141 nM versus 262 nM), with larger sample sizes required for the remaining bile acids. Despite these power and detection limitations, we observed lower mean CA, GUDCA, and GHCA concentrations with lignan supplementation. Further, we analyzed bile acids in fasting plasma. Given the homeostatic regulation of bile acids, we may not have adequately captured intervention effects that may have occurred post-prandially. We also did not measure sulfated bile acid species. Sulfation is an important elimination pathway and contributes to bile acid homeostasis [72,73].

Dietary factors such as fat and fiber intake play a role in determining bile acid concentrations [74,75]; these were not controlled in this study. As we recruited based on lower fiber intake, it is unlikely to be a factor. While fat intake was lower among high-ENL excreters, considering the crossover study design, this is also unlikely to contribute to our results, although it may have increased the variability across individuals. Nonetheless, we conducted a sensitivity analyses adjusting for dietary fat in the statistical models and found that point estimates were no different with or without adjustment. Our study lacks fecal bile acid measures for comparison with plasma bile acid concentrations. Plasma bile acid concentrations have been shown to reflect hepatic concentrations [76], however, it is not known how they relate to concentrations in the gut lumen. FXR is widely distributed in various tissues, including liver, intestine, and adipose, and it has been proposed that circulating bile acids are required for maintaining FXR expression [77].

Regarding the exploratory analysis, in the intervention we were only able to evaluate associations between ENL excretion and bile acids with gut bacteria at the genera level. Our in vitro experiments and work by Heinken et al. [39] suggest that there are strain-level differences in metabolism and our analysis using 16S rRNA may not have provided sufficient detail. While our in vitro incubations were in a small subset of samples, they did show overlap in bacterial species that produce both ENL and secondary bile acids, and that ENL production was associated with gene expression in secondary bile acid pathways. Future application of this in vitro approach should use combinatorial incubations with both SDG and bile acids to understand the impact of ENL metabolic mechanisms on secondary bile acid speciation that may impact host exposures.

## 5. Conclusions

In conclusion, we found modest intervention effects of a flaxseed lignan extract on circulating CA and its conjugates, overall and by ENL excreter status. In the cross-sectional analysis we observed robust associations between ENL excretion and certain circulating secondary bile acids, particularly secondary CA-derived species, possibly reflecting lower CA available as substrate. Our in vitro approach supports the application of metagenomic and metatranscriptomics to reveal the gene abundance and expression of species and genes involved in ENL and bile acid metabolism. Analysis of these microbial pathways within a larger controlled dietary intervention can provide the analytical rigor needed to unravel the impact of ENL on bile acid metabolism and host health.

## Figures and Tables

**Table 1:** Enzymes involved in secondary bile acid synthesis.

Abbreviation	Enzyme
BSH	Choloylglycine Hydrolase (EC 3.5.1.24)
12aHSDH	12–alpha-hydroxysteroid dehydrogenase (EC 1.1.1.176)
7aHSDH	7–alpha-hydroxysteroid dehydrogenase (EC 1.1.1.159)
7bHSDH	7–beta-hydroxysteroid dehydrogenase (EC 1.1.1.201)
3aHSDH	3–alpha-hydroxysteroid dehydrogenase (EC 1.1.1.53)
3bHSDH	3–beta-hydroxysteroid dehydrogenase (EC 1.1.1.145
Bile acid inducible (Bai) Gene Cluster	
BaiA	NAD(P)-dependent 3–alpha-hydroxysteroid dehydrogenase (EC 1.1.1.50)
BaiB	Bile acid-coenzyme A ligase (EC 6.2.1.7)
BaiCD	NAD(H)-dependent 7–alpha-hydroxy–3–oxo-delta 4–cholenoic acid oxidoreductase
BaiE	Bile acid 7–alpha dehydratase (EC 4.2.1.106)
BaiF	Bile acid CoA-transferase (EC 2.8.3.25)
BaiG	Bile acids transporter, MFS family
BaiH	NAD(H)-dependent 7–beta-hydroxy–3–oxo-delta 4–cholenoic acid oxidoreductase
BaiI	Bile acid 7–beta-dehydratase (EC 4.2.1.106)
CHSTR	Cholesterol reductase (gene not known)

**Table 2:** Characteristics of participants in the FlaxFX intervention study overall and stratified by ENL excretion ^a^.

Characteristic	All Participants	Low ENL	High ENL (*n* = 23)
	(*n* = 46)	(*n* = 23)	
Female *n* (%)	23 (50.0)	11 (47.8)	12 (52.2)
Age (years)	32.1 (8.4)	33.1 (9.3)	31.1 (7.6)
BMI (kg/m^2^)	26.7 (5.8)	27.2 (7.1)	26.2 (4.3)
Energy intake (kcal/day) ^b^	2067 (518)	2289 (602)	1865 (324)
Dietary fat (g/day) ^b^	86 (30)	96 (34)	77 (23)
Dietary fiber (g/day) ^b^	20.5 (7)	19.2 (7.2)	21.6 (6.7)
Dietary protein (g/day) ^b^	86.9 (30.3)	99.8 (35.5)	74.6 (17.6)
Race/ethnicity (n)			
American Indian	2	1	1
Asian	11	7	4
Black African American	2	1	1
Caucasian	28	12	16
Other (more than 1 race)	3	2	1
Urinary Enterolignans (µmol/24 h) ^c^			
SECO	0.4 (0.5)	0.5 (0.6)	0.3 (0.4)
END	1.1 (2.7)	1.2 (3)	1.1 (2.4)
ENL	7.6 (14.3)	4.1 (5)	11.1 (19.2)
Baseline plasma bile acid groups (nM) ^d^			
Σ Total bile acids	2630 (2515)	2623 (2652)	2639 (2428)
Σ Primary bile acids	1511 (1747)	1411 (1633)	1617 (1890)
Σ Secondary bile acids	1119 (984)	1212 (1173)	1022 (737)
Σ Total taurine-conjugated	293 (666)	193.6 (200)	397 (926)
Σ Total glycine-conjugated	1309 (1274)	1284 (1103)	1336 (1455)

^a^ All values are baseline measures presented as means (SD) unless otherwise indicated. Low and high enterolactone (ENL) defined by below and above median urinary excretion after the lignan flaxseed intervention period (22.1 µmol/24 h). ^b^ Based on 3–day food records. *N* = 44 (*n* = 2 excluded based on biologically implausible energy intakes). ^c^ Values at the end of the placebo intervention period; zeros imputed as half the lowest value for each variable. ^d^ One person with high baseline bile acid concentrations omitted from bile acid totals. BMI, body mass index; SECO, secoisolariciresinol; END, enterodiol; ENL, enterolactone.

**Table 3:** Effects of a flaxseed lignan supplement compared to placebo overall and stratified by ENL excretion on plasma bile acids in a randomized, controlled, crossover intervention ^a^.

Bile Acid	All Participants (*n* = 45)			Low ENL (*n* = 23)			High ENL (*n* = 22)		
	Placebo	Flax	*P* ^b^	Placebo	Flax	*P* ^b^	Placebo	Flax	*P* ^b^
Primary									
Σ Primary bile acids	1364 (960)	1202 (868)	0.18	1415 (865)	1337 (899)	0.72	1310 (1067)	1060 (830)	0.25
Cholic acid	262 (313)	142 (88)	0.004	275 (379)	134 (60)	0.04	284 (233)	149 (110)	0.04
Taurohyocholic acid	6.5 (4.0)	5.8 (3.9)	0.14	5.9 (3.3)	5.2 (2.4)	0.51	7.3 (4.6)	6.4 (5.1)	0.13
Glycocholic acid	273 (306)	269 (309)	0.62	234 (169)	286 (320)	0.94	315 (403)	252 (304)	0.44
Taurocholic acid	123 (188)	132 (182)	0.64	88.7 (58.8)	116 (137)	0.92	159 (250)	150 (220)	0.52
Chenodeoxycholic acid	430 (420)	388 (386)	0.85	518 (485)	501 (474)	0.96	338 (324)	270 (219)	0.84
Glycochenodeoxycholic acid	269 (217)	265 (232)	0.96	293 (224)	296 (234)	0.94	243 (212)	233 (231)	0.99
Secondary									
Σ Secondary bile acids	1830 (726)	1802 (900)	0.34	1900 (819)	1961 (990)	0.51	1756 (626)	1635 (782)	0.91
Deoxycholic acid	480 (338)	453 (318)	0.86	486 (371)	487 (326)	0.49	473 (308)	417 (314)	0.23
Taurodeoxycholic acid	57.5 (62.2)	65.5 (84.5)	0.90	60.3 (71.8)	63.5 (88.8)	0.89	55.6 (51.8)	67.5 (81.8)	0.77
Glycodeoxycholic acid	311 (295)	311 (304)	0.72	344 (331)	331 (324)	0.84	275 (253)	290 (288)	0.75
Lithocholic acid	73.3 (40.1)	72.2 (49.6)	0.87	66.8 (45.2)	72.6 (46.1)	0.38	80.1 (33.5)	71.7 (32.4)	0.29
Glycolithocholic acid	69.4 (39.4)	65.0 (37.3)	0.63	70.1 (46.0)	66.5 (45.1)	0.90	68.6 (32.1)	63.4 (27.8)	0.54
Taurolithocholic acid	14.9 (7.0)	18.3 (15.1)	0.24	13.1 (6.8)	17.0 (9.1)	0.01	16.7 (6.8)	19.6 (19.7)	0.58
Isolithocholic acid	73.5 (33.0)	73.8 (32.6)	0.97	59.8 (24.2)	68.7 (36.3)	0.29	87.9 (35.4)	79.2 (28.1)	0.28
Glycoursodeoxycholic acid	146 (104)	134 (108)	0.17	169 (122)	155 (112)	0.82	122 (76)	112 (103)	0.05
Glycohyodeoxycholic acid	132 (91)	122 (90)	0.33	150 (105)	139 (92)	0.99	113 (69)	105 (87)	0.14
Hyodeoxycholic acid	106 (48)	107 (68)	0.46	108 (50)	124 (88)	0.96	104 (47)	77.8 (31.5)	0.16
Glycohyocholic acid	36.3 (27.4)	29.9 (25.1)	0.11	33.7 (28.6)	30.6 (29.6)	0.60	39.0 (26.4)	29.3 (20.0	0.03
5β-Cholanic acid-3β, 12α-diol	158 (81)	167 (102)	0.61	160 (79)	191 (117)	0.21	156 (85)	142 (81)	0.61
Muricholic acid	164 (124)	176 (172)	0.78	171 9137)	208 (202)	0.28	158 (111)	143 (131)	0.22
Tauro-α-muricholic acid	8.1 (9.6)	6.9 (8.7)	0.08	8.3 (10.5)	6.6 (9.8)	0.07	7.7 (8.8)	7.3 (7.6)	0.46
Σ Glycine-conjugated bile acids	1237 (925)	1196 (925)	0.62	1295 (876)	1304 (967)	0.95	1176 (860)	1084 (887)	0.15
Σ Taurine-conjugated bile acids	210 (231)	229 (260)	0.96	176 (134)	208 (228)	0.94	245 (301)	251 (293)	0.95

^a^ Data presented as absolute nM concentrations (SD). Low and high enterolactone (ENL) defined by below and above median urinary excretion after the lignan flaxseed intervention period (22.1 µmol/24 h). ^b^ Raw *P* values generated from linear mixed models evaluating the effects of intervention on bile acids, adjusted for age, sex, body mass index, treatment sequence, baseline values, and batch. No intervention effects were significant at false discovery rate < 0.05. One outlier with high baseline values was excluded from these intervention analyses.

**Table 4:** Cross-sectional association of plasma bile acids and enterolactone (ENL) excretion irrespective of intervention (µmol/24 h urine; *n* = 46).

Bile Acid	Type	β	SE	*P* Value ^a^
Glycoursodeoxycholic acid	Secondary	−0.2144	0.0455	2.39 × 10^−6^ *
Glycohyodeoxycholic acid	Secondary	−0.1964	0.0439	7.69 × 10^−6^ *
Isolithocholic acid	Secondary	0.1095	0.0298	0.0002 *
Hyodeoxycholic acid	Secondary	−0.1095	0.0298	0.0002 *
Muricholic acid	Secondary	−0.1715	0.0513	0.0008 *
Lithocholic acid	Secondary	0.0999	0.0397	0.01
Chenodeoxycholic acid	Primary	−0.1359	0.0628	0.03
Glycolithocholic acid	Secondary	0.0654	0.0388	0.09
Taurolithocholic acid	Secondary	0.0786	0.0434	0.07
Cholic acid	Primary	0.0685	0.0475	0.15
5β-Cholanic acid-3β, 12α-diol	Secondary	−0.0431	0.0366	0.24
Glycochenodeoxycholic acid	Primary	−0.0701	0.0674	0.30
Taurodeoxycholic acid	Secondary	0.0746	0.0815	0.36
Deoxycholic acid	Secondary	0.0473	0.0537	0.38
Tauro-α-muricholic acid	Secondary	0.0418	0.0825	0.61
Glycocholic acid	Primary	−0.0477	0.0759	0.53
Glycodeoxycholic acid	Secondary	0.0256	0.0745	0.73
Glycohyocholic acid	Secondary	−0.0085	0.0496	0.86
Taurocholic acid	Primary	−0.0044	0.0773	0.95
Taurohyocholic acid	Primary	0.0001	0.0416	1.00

^a^ Linear mixed model evaluating the association of bile acids and ENL excretion (continuous), adjusted for age, sex, body mass index, and batch for both time-points per person (*n* = 96 observations). * Statistically significant with false discovery rate < 0.05.

## Supplementary Materials

The following are available online at https://www.mdpi.com/2072-6643/12/6/1837/s1, Figure S1: CONSORT diagram; Table S1: TCAP2 Media components for in vitro incubations; Table S2: Association between gut bacterial genera at the end of placebo and ENL excretion and plasma bile acids after 60 days of flaxseed lignan intervention (*n* = 44) ^a^.

## References

[1] McRae M.P. (2017). Health benefits of dietary whole grains: An umbrella review of meta-analyses. J. Chiropr. Med..

[2] Bradbury K.E., Appleby P.N., Key T.J. (2014). Fruit, vegetable, and fiber intake in relation to cancer risk: Findings from the European Prospective Investigation into Cancer and Nutrition (EPIC). Am. J. Clin. Nutr..

[3] Kahleova H., Levin S., Barnard N. (2017). Cardio-metabolic benefits of plant-based diets. Nutrients.

[4] Bamia C. (2018). Dietary patterns in association to cancer incidence and survival: Concept, current evidence, and suggestions for future research. Eur. J. Clin. Nutr..

[5] Tangestani H., Emamat H., Ghalandari H., Shab-Bidar S. (2020). Whole grains, dietary fibers and the human gut microbiota: A systematic review of existing literature. Recent Pat Food Nutr. Agric..

[6] Wang L., Meselhy M.R., LI Y., Qin G., Hattori M. (2000). Human intestinal bacteria capable of transforming secoisolariciresinol diglucoside to mammalian lignans, enterodiol and enterolactone. Chem. Pharmol. Bull..

[7] Webb A.L., McCullough M.L. (2005). Dietary lignans: Potential role in cancer prevention. Nutr. Cancer.

[8] Kuijsten A., Arts I.C., Vree T.B., Hollman P.C. (2005). Pharmacokinetics of enterolignans in healthy men and women consuming a single dose of secoisolariciresinol diglucoside. J. Nutr..

[9] Miles F.L., Navarro S.L., Schwarz Y., Gu H., Djukovic D., Randolph T.W., Shojaie A., Kratz M., Hullar M.A.J., Lampe P.D. (2017). Plasma metabolite abundances are associated with urinary enterolactone excretion in healthy participants on controlled diets. Food Funct..

[10] Chiang J.Y.L. (2017). Targeting bile acids and lipotoxicity for NASH treatment. Hepatol. Commun..

[11] Ajouz H., Mukherji D., Shamseddine A. (2014). Secondary bile acids: An underrecognized cause of colon cancer. World J. Surg. Oncol..

[12] Ding L., Yang L., Wang Z., Huang W. (2015). Bile acid nuclear receptor FXR and digestive system diseases. Acta Pharm. Sin. B.

[13] Chiang J.Y.L., Ferrell J.M. (2019). Bile Acids as Metabolic Regulators and Nutrient Sensors. Annu. Rev. Nutr..

[14] Ding L., Pang S., Sun Y., Tian Y., Yu L., Dang N. (2014). Coordinated actions of FXR and LXR in metabolism: From pathogenesis to pharmacological targets for Type 2 Diabetes. Int. J. Endocrinol..

[15] Batta A.K., Aggarwal S.K., Salen G., Shefer S. (1991). Selective reduction of oxo bile acids: Synthesis of 3 beta-, 7 beta-, and 12 beta-hydroxy bile acids. J. Lipid Res..

[16] Lampe J.W., Kim E., Levy L., Davidson L.A., Goldsby J.S., Miles F.L., Navarro S.L., Randolph T.W., Zhao N., Ivanov I. (2019). Colonic mucosal and exfoliome transcriptomic profiling and fecal microbiome response to a flaxseed lignan extract intervention in humans. Am. J. Clin. Nutr..

[17] Block G., Gillespie C., Rosenbaum E.H., Jenson C. (2000). A rapid food screener to assess fat and fruit and vegetable intake. Am. J. Prev. Med..

[18] Patterson R.E., Kristal A.R., Tinker L.F., Carter R.A., Bolton M.P., Agurs-Collins T. (1999). Measurement characteristics of the Women’s Health Initiative food frequency questionnaire. Ann. Epidemiol..

[19] Peñalvo J.L., Haajanen K.M., Botting N., Adlercreutz H. (2005). Quantification of lignans in food using isotope dilution gas chromatography/mass spectrometry. J. Agric. Food Chem..

[20] Ginos B.N.R., Navarro S.L., Schwarz Y., Gu H., Wang D., Randolph T.W., Shojaie A., Hullar M.A.J., Lampe P.D., Kratz M. (2018). Circulating bile acids in healthy adults respond differently to a dietary pattern characterized by whole grains, legumes and fruits and vegetables compared to a diet high in refined grains and added sugars: A randomized, controlled, crossover feeding study. Metabolism.

[21] MacLean B., Tomazela D.M., Shulman N., Chambers M., Finney G.L., Frewen B., Kern R., Tabb D.L., Liebler D.C., MacCoss M.J. (2010). Skyline: An open source document editor for creating and analyzing targeted proteomics experiments. Bioinformatics.

[22] Raftery D. (2014). Mass Spectrometry in Metabolomics: Methods and Protocols.

[23] Sarafian M.H., Lewis M.R., Pechlivanis A., Ralphs S., McPhail M.J., Patel V.C., Dumas M.E., Holmes E., Nicholson J.K. (2015). Bile acid profiling and quantification in biofluids using ultra-performance liquid chromatography tandem mass spectrometry. Anal. Chem..

[24] Fu B.C., Randolph T.W., Lim U., Monroe K.R., Cheng I., Wilkens L.R., Le Marchand L., Lampe J.W., Hullar M.A.J. (2019). Temporal variability and stability of the fecal microbiome: The Multiethnic Cohort Study. Cancer Epidemiol. Biomarkers Prev..

[25] Satinsky B.M., Gifford S.M., Crump B.C., Moran M.A. (2013). Use of internal standards for quantitative metatranscriptome and metagenome analysis. Methods Enzymol..

[26] Stewart F.J., Ottesen E.A., DeLong E.F. (2010). Development and quantitative analyses of a universal rRNA-subtraction protocol for microbial metatranscriptomics. ISME J..

[27] Li F., Hullar M.A., Lampe J.W. (2007). Optimization of terminal restriction fragment polymorphism (TRFLP) analysis of human gut microbiota. J. Microbiol. Methods.

[28] Zoetendal E.G., Heilig H.G., Klaassens E.S., Booijink C.C., Kleerebezem M., Smidt H., de Vos W.M. (2006). Isolation of DNA from bacterial samples of the human gastrointestinal tract. Nat. Protoc..

[29] El Fantroussi S., Urakawa H., Bernhard A.E., Kelly J.J., Noble P.A., Smidt H., Yershov G.M., Stahl D.A. (2003). Direct profiling of environmental microbial populations by thermal dissociation analysis of native rRNAs hybridized to oligonucleotide microarrays. Appl. Environ. Microb..

[30] Baker G.C., Smith J.J., Cowan D.A. (2003). Review and re-analysis of domain-specific 16S primers. J. Microbiol. Methods.

[31] Rognes T., Flouri T., Nichols B., Quince C., Mahe F. (2016). VSEARCH: A versatile open source tool for metagenomics. PeerJ.

[32] Caporaso J.G., Kuczynski J., Stombaugh J., Bittinger K., Bushman F.D., Costello E.K., Fierer N., Pena A.G., Goodrich J.K., Gordon J.I. (2010). QIIME allows analysis of high-throughput community sequencing data. Nat. Methods.

[33] Bolger A.M., Lohse M., Usadel B. (2014). Trimmomatic: A flexible trimmer for Illumina sequence data. Bioinformatics.

[34] Abubucker S., Segata N., Goll J., Schubert A.M., Izard J., Cantarel B.L., Rodriguez-Mueller B., Zucker J., Thiagarajan M., Henrissat B. (2012). Metabolic reconstruction for metagenomic data and its application to the human microbiome. PLoS Comput. Biol..

[35] Buchfink B., Xie C., Huson D.H. (2015). Fast and sensitive protein alignment using DIAMOND. Nat. Methods.

[36] Suzek B.E., Wang Y.Q., Huang H.Z., McGarvey P.B., Wu C.H., Consortium U. (2015). UniRef clusters: A comprehensive and scalable alternative for improving sequence similarity searches. Bioinformatics.

[37] Caspi R., Billington R., Ferrer L., Foerster H., Fulcher C.A., Keseler I.M., Kothari A., Krummenacker M., Latendresse M., Mueller L.A. (2016). The MetaCyc database of metabolic pathways and enzymes and the BioCyc collection of pathway/genome databases. Nucleic Acids Res..

[38] Benjamini Y., Hochberg Y. (1995). Controlling the false discovery rate—A practical and powerful approach to multiple testing. J. R. Stat. Soc. B Met..

[39] Heinken A., Ravcheev D.A., Baldini F., Heirendt L., Fleming R.M.T., Thiele I. (2019). Systematic assessment of secondary bile acid metabolism in gut microbes reveals distinct metabolic capabilities in inflammatory bowel disease. Microbiome.

[40] Touré A., Xueming X. (2010). Flaxseed lignans: Source, biosynthesis, metabolism, antioxidant activity, bio-active components, and health benefits. Compr. Rev. Food Sci. Food Saf..

[41] Gloor G.B., Macklaim J.M., Pawlowsky-Glahn V., Egozcue J.J. (2017). Microbiome datasets are compositional: And this is not optional. Front. Microbiol..

[42] Zeng H., Jiang Y., Chen P., Fan X., Li D., Liu A., Ma X., Xie W., Liu P., Gonzalez F.J. (2017). Schisandrol B protects against cholestatic liver injury through pregnane X receptors. Br. J. Pharmacol..

[43] Fan S., Liu C., Jiang Y., Gao Y., Chen Y., Fu K., Yao X., Huang M., Bi H. (2019). Lignans from Schisandra sphenanthera protect against lithocholic acid-induced cholestasis by pregnane X receptor activation in mice. J. Ethnopharmacol..

[44] Cao W.R., Ge J.Q., Xie X., Fan M.L., Fan X.D., Wang H., Dong Z.Y., Liao Z.H., Lan X.Z., Chen M. (2017). Protective effects of petroleum ether extracts of Herpetospermum caudigerum against alpha-naphthylisothiocyanate-induced acute cholestasis of rats. J. Ethnopharmacol..

[45] Bohmdorfer M., Maier-Salamon A., Taferner B., Reznicek G., Thalhammer T., Hering S., Hufner A., Schuhly W., Jager W. (2011). In Vitro metabolism and disposition of honokiol in rat and human livers. J. Pharm. Sci..

[46] Takeda S., Arai I., Hasegawa M., Tatsugi A., Aburada M., Hosoya E. (1988). [Effect of gomisin A (TJN-101), a lignan compound isolated from Schisandra fruits, on liver function in rats]. Nihon Yakurigaku Zasshi.

[47] Ohtaki Y., Hida T., Hiramatsu K., Kanitani M., Ohshima T., Nomura M., Wakita H., Aburada M., Miyamoto K.I. (1996). Deoxycholic acid as an endogenous risk factor for hepatocarcinogenesis and effects of gomisin A, a lignan component of Schizandra fruits. Anticancer Res..

[48] Miyamoto K., Hiramatsu K., Ohtaki Y., Kanitani M., Nomura M., Aburada M. (1995). Effects of gomisin A on the promotor action and serum bile acid concentration in hepatocarcinogenesis induced by 3′-methyl-4-dimethylamino-azobenzene. Biol. Pharm. Bull..

[49] Sanghvi A., Diven W.F., Seltman H.J., Paul R., Rizk M. (1984). Properties of cholesterol 7 alpha-hydroxylase in rat liver microsomal acetone powder. Metabolism.

[50] Tai T.S., Tien N., Shen H.Y., Chu F.Y., Wang C.C.N., Lu C.H., Yu H.I., Kung F.P., Chuang H.H., Lee Y.R. (2019). Sesamin, a Naturally Occurring Lignan, Inhibits Ligand-Induced Lipogenesis through Interaction with Liver X Receptor Alpha (LXRalpha) and Pregnane X Receptor (PXR). Evid. Based Complement. Altern. Med..

[51] Hirose N., Inoue T., Nishihara K., Sugano M., Akimoto K., Shimizu S., Yamada H. (1991). Inhibition of cholesterol absorption and synthesis in rats by sesamin. J. Lipid Res..

[52] Jacobs M.N., Nolan G.T., Hood S.R. (2005). Lignans, bacteriocides and organochlorine compounds activate the human pregnane X receptor (PXR). Toxicol. Appl. Pharmacol..

[53] Chen J., Zhao K.N., Chen C. (2014). The role of CYP3A4 in the biotransformation of bile acids and therapeutic implication for cholestasis. Ann. Transl. Med..

[54] Kim H., Fang S. (2018). Crosstalk between FXR and TGR5 controls glucagon-like peptide 1 secretion to maintain glycemic homeostasis. Lab. Anim. Res..

[55] Perez M.J., Briz O. (2009). Bile-acid-induced cell injury and protection. World J. Gastroenterol..

[56] Chiang J.Y. (2009). Bile acids: Regulation of synthesis. J. Lipid Res..

[57] Ege T., Gençler-Özkan A.M., Şen A., Adalı O. (2018). Effects of folk medicinal plant epilobium hirsutum l. And its ingredient ellagic acid on rat liver bile acid synthesizing cyps in rats. PharmacologyOnline.

[58] Russell D.W. (2003). The enzymes, regulation, and genetics of bile acid synthesis. Annu. Rev. Biochem..

[59] Ridlon J.M., Kang D.J., Hylemon P.B. (2006). Bile salt biotransformations by human intestinal bacteria. J. Lipid Res..

[60] Dawson P.A., Karpen S.J. (2015). Intestinal transport and metabolism of bile acids. J. Lipid Res..

[61] Zhang J., Gao L.Z., Chen Y.J., Zhu P.P., Yin S.S., Su M.M., Ni Y., Miao J., Wu W.L., Chen H. (2019). Continuum of host-gut microbial Co-metabolism: Host CYP3A4/3A7 are responsible for tertiary Oxidations of deoxycholate species. Drug Metab. Dispos..

[62] Lin Q., Tan X., Wang W., Zeng W., Gui L., Su M., Liu C., Jia W., Xu L., Lan K. (2020). Species differences of bile acid redox metabolism: Tertiary oxidation of deoxycholate is conserved in preclinical animals. Drug Metab. Dispos..

[63] Ding Y., Yanagi K., Cheng C., Alaniz R.C., Lee K., Jayaraman A. (2019). Interactions between gut microbiota and non-alcoholic liver disease: The role of microbiota-derived metabolites. Pharmacol. Res..

[64] Nihira T., Suzuki E., Kitaoka M., Nishimoto M., Ohtsubo K., Nakai H. (2013). Discovery of beta-1,4-D-mannosyl-N-acetyl-D-glucosamine phosphorylase involved in the metabolism of N-glycans. J. Biol. Chem..

[65] Yoder S., Lancaster S., Hullar M.A.J., Lampe J.W., Del Rio D., Tuohy K. (2015). Gut microbial metabolism of plant lignans: Influence on human health. Diet-Microbe Interactions in the Gut.

[66] Halldin E., Eriksen A.K., Brunius C., da Silva A.B., Bronze M., Hanhineva K., Aura A.M., Landberg R. (2019). Factors explaining inter-personal variation in plasma enterolactone concentrations in humans. Mol. Nutr. Food Res..

[67] Liu N., Fosses A., Kampik C., Parsiegla G., Denis Y., Vita N., Fierobe H.P., Perret S. (2019). In Vitro and In Vivo exploration of the cellobiose and cellodextrin phosphorylases panel in Ruminiclostridium cellulolyticum: Implication for cellulose catabolism. Biotechnol. Biofuels.

[68] Ren Z., You W., Wu S., Poetsch A., Xu C. (2019). Secretomic analyses of Ruminiclostridium papyrosolvens reveal its enzymatic basis for lignocellulose degradation. Biotechnol. Biofuels.

[69] Harris S.C., Devendran S., Mendez-Garcia C., Mythen S.M., Wright C.L., Fields C.J., Hernandez A.G., Cann I., Hylemon P.B., Ridlon J.M. (2018). Bile acid oxidation by Eggerthella lenta strains C592 and DSM 2243(T). Gut Microbes.

[70] Bess E.N., Bisanz J.E., Yarza F., Bustion A., Rich B.E., Li X., Kitamura S., Waligurski E., Ang Q.Y., Alba D.L. (2020). Genetic basis for the cooperative bioactivation of plant lignans by Eggerthella lenta and other human gut bacteria. Nat. Microbiol..

[71] Song Z., Cai Y., Lao X., Wang X., Lin X., Cui Y., Kalavagunta P.K., Liao J., Jin L., Shang J. (2019). Taxonomic profiling and populational patterns of bacterial bile salt hydrolase (BSH) genes based on worldwide human gut microbiome. Microbiome.

[72] Thakare R., Alamoudi J.A., Gautam N., Rodrigues A.D., Alnouti Y. (2018). Species differences in bile acids I. Plasma and urine bile acid composition. J. Appl. Toxicol..

[73] Alnouti Y. (2009). Bile Acid sulfation: A pathway of bile acid elimination and detoxification. Toxicol. Sci..

[74] Ghaffarzadegan T., Zanzer Y.C., Ostman E., Hallenius F., Essen S., Sandahl M., Nyman M. (2019). Postprandial responses of serum bile acids in healthy humans after ingestion of turmeric before medium/high-fat breakfasts. Mol. Nutr. Food Res..

[75] Story J.A., Furumoto E.J., Buhman K.K. (1997). Dietary fiber and bile acid metabolism—An update. Adv. Exp. Med. Biol..

[76] Luo L., Aubrecht J., Li D., Warner R.L., Johnson K.J., Kenny J., Colangelo J.L. (2018). Assessment of serum bile acid profiles as biomarkers of liver injury and liver disease in humans. PLoS ONE.

[77] Xu G., Pan L.X., Li H., Forman B.M., Erickson S.K., Shefer S., Bollineni J., Batta A.K., Christie J., Wang T.H. (2002). Regulation of the farnesoid X receptor (FXR) by bile acid flux in rabbits. J. Biol. Chem..

