# Supplementary material for: Effect of a Flaxseed Lignan Intervention on Circulating Bile Acids in a Placebo-Controlled Randomized, Crossover Trial
Source: Nutrients. 2020 Jun 19;12(6):1837. doi: 10.3390/nu12061837 (PMC7374341; doi:10.3390/nu12061837)
Supplement: Supplementary file 1 [file nutrients-12-01837-s001.pdf]

Figure S1. Study design.

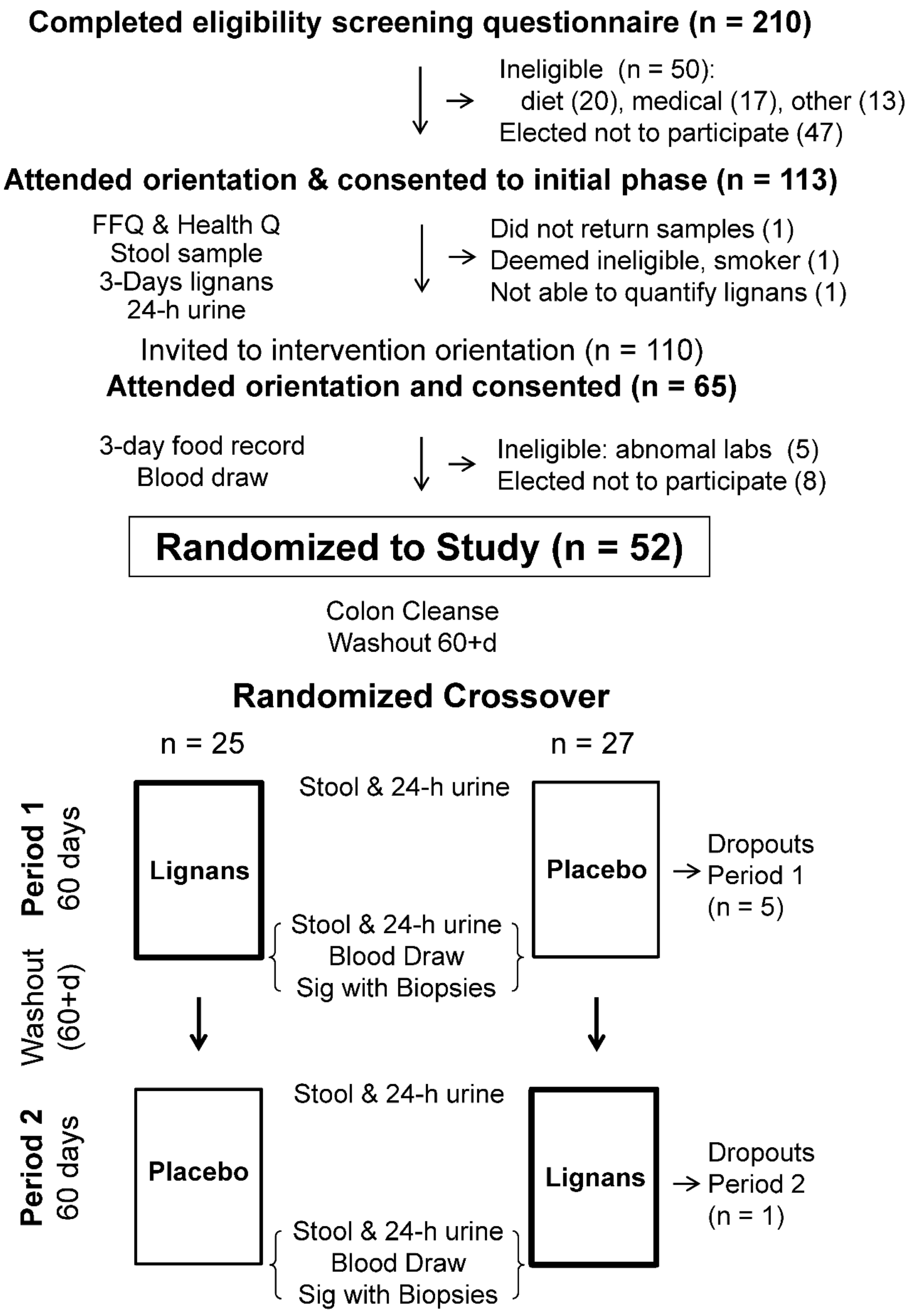

Table S1. TCAP2 Media Components for *in vitro* incubations.

Reagent	g/L
L-Cysteine-HCl (Aldrich)	0.25
Glucose (Sigma)	0.9
Resazurin (1mg/L) (Aldrich)	1
NaCl (Sigma)	2.78
K2HPO4 (Sigma)	0.42
Na2HPO4·7H2O (Fisher)	3.3
NaH2PO4·2H2O (Fluka)	1.2
(NH4)2SO4 (Fisher)	1.2
MgCl2·6H2O (Sigma)	0.08
KH2PO4 (Fisher)	0.16
CH3COONa (Fisher)	0.5
HCOONa (Fluka)	0.5
Trace element solution*	10 ml
Vitamin solution**	1 ml

***Trace Element Solution mg/L**

**Reagent**	**mg/L**
MnSO4·H2O (Fisher)	1159
FeSO4·7H2O (Fisher)	3680
CoCl2·6H2O (MP Biomedicals)	120
ZnSO4·7H2O (Fisher)	440
CuSO4·5H2O (Fisher)	98
(NH4)6Mo7O24·4H2O (Fluka)	17.4

****Vitamin Solution mg/L**

**Reagent**	**mg/L**
Menadione (MP Biomedicals)	1
Biotin (Jackson Immuno Research)	2
Pantothenate (MP Biomedicals)	10
Nicotinamide (Acros)	5
Vitamin B12 (MP Biomedicals)	0.5
Thiamine (MP Biomedicals)	4
ρ-Aminobenzoic acid (MP Biomedicals)	5

Table S2. Association between gut bacterial genera at the end of placebo and ENL excretion and plasma bile acids after 60 days of flaxseed lignan intervention (n=44)[a].

**Bacterial Taxon**		**Primary Bile Acids**						**Secondary Bile Acids**									
	**ENL**	Cholic Acid	Taurocholic Acid	Glycocholic Acid	Taurohyocholic Acid	Chenodeoxycholic Acid	Glycochenodeoxycholic Acid	Lithocholic Acid	Glycolithocholic Acid	Taurolithocholic Acid	Isolithocholic Acid	Glycoursodeoxycholic Acid	Glycohyodeoxycholic Acid	Hyodeoxycholic Acid	Glycohyocholic Acid	Muricholic Acid	Tauro-Alpha-Muricholic Acid
**Actinobacteria;__ Coriobacteriia**																	
Coriobacteriales_Incertae_Sedis;__uncultured																	
Coriobacteriales;__Eggerthellaceae;__Senegalimassilia																	
Coriobacteriales;__Eggerthellaceae;__Slackia																	
Bacteroidetes;__ Bacteroidia;__ Bacteroidales																	
Bacteroidaceae;__Bacteroides																	
Bacteroidales_RF16_group;__uncultured_bacterium																	
Muribaculaceae;__uncultured_bacterium																	
Prevotellaceae;__Alloprevotella																	
Rikenellaceae;__Alistipes																	
**Cyanobacteria;__ Melainabacteria**																	
Gastranaerophilales;__gut_metagenome;Other																	
**Firmicutes;__ Clostridia;__ Clostridiales**																	
Other;Other																	
Christensenellaceae;__Christensenellaceae_R-																	
Clostridiales_vadinBB60_grp;__uncultured_organism																	
Lachnospiraceae;__Blautia																	
Lachnospiraceae;__Coprococcus_3																	
Lachnospiraceae;__Lachnospiraceae_UCG-003																	
Lachnospiraceae;__Lachnospiraceae_UCG-004																	
Lachnospiraceae;__Lachnospiraceae_UCG-008																	
Lachnospiraceae;__Tyzzerella_3																	
Lachnospiraceae;__[Eubacterium]_hallii_group																	
Lachnospiraceae;__[Eubacterium]_ruminantium_grou																	
Lachnospiraceae;__[Eubacterium]_ventriosum_group																	
Lachnospiraceae;__uncultured																	
Ruminococcaceae;__Butyricicoccus																	
Ruminococcaceae;__Faecalibacterium																	
Ruminococcaceae;__Flavonifractor																	
Ruminococcaceae;__Ruminiclostridium_5																	
Ruminococcaceae;__Ruminiclostridium_6*												*	*				
Ruminococcaceae;__Ruminiclostridium_9																	
Ruminococcaceae;__Ruminococcaceae_NK4A214_grp																	
Ruminococcaceae;__Ruminococcaceae_UCG-010																	

Ruminococcaceae;__Ruminococcaceae_UCG-013
Ruminococcaceae;__Ruminococcaceae_UCG-014
Ruminococcaceae;__Ruminococcus_2
Ruminococcaceae;__UBA1819
**Firmicutes;__ Erysipelotrichia;__ Erysipelotrichales**
Erysipelotrichaceae;__Catenibacterium
Erysipelotrichaceae;__Erysipelatoclostridium
Erysipelotrichaceae;__Firmicutes_bacterium_CAG:34
Lentisphaerae;__ Lentisphaeria
Victivallales;__vadinBE97;Other
**Proteobacteria;__ Alphaproteobacteria**
Rhodospirillales;__uncultured;__gut_metagenome
Rhodospirillales;__uncultured;__uncultured_bacteriu
**Proteobacteria;__ Gammaproteobacteria**
Pasteurellales;__Pasteurellaceae;__Aggregatibacter
Pasteurellales;__Pasteurellaceae;__Haemophilus
**Tenericutes;__ Mollicutes**
Mollicutes_RF39;__gut_metagenome;Other
Mollicutes_RF39;__uncultured_bacterium;Other

[a]Results derived from linear mixed models evaluating the association between genera and ENL excretion and bile acids separately, adjusted for age, sex, body mass index, treatment, and batch for bile acids. 147 genera were tested and only those with raw *P* values less than 0.01 are shown; values in blue represent negative coefficients while orange represents positive coefficients. An asterisk indicates FDR<0.1. The secondary bile acids: deoxycholic acid, taurodeoxycholic acid, glycodeoxycholic acid and beta-cholanic acid-3beta, 12alpha-diol were not associated with any bacteria. Microbial DNA extracted from two participants did not pass quality control.
